# Analysis of Urinary Flora Characteristics in Urinary Tumor Based on 16S rRNA Sequence

**DOI:** 10.1155/2022/9368687

**Published:** 2022-07-14

**Authors:** Jin Qiu, Jianxin Liu, Yi Zhong, Wang Liu, Zhangjie Zhou, Yun Li, Shiying Li

**Affiliations:** Shanghai TCM-Integrated Hospital, Shanghai University of Chinese Medicine, Shanghai 200082, China

## Abstract

The relationship between urinary system tumors and urothelial microorganisms remains unexplored. This study is aimed at exploring the relationship between urinary flora and urinary tumors and identifying potential biomarkers for urinary tumors and new targets for prevention. We included four healthy adults (control group) and six patients diagnosed with urinary tract tumors (tumor group). In both groups, 10 and 50 ml clean middle urine samples were reserved. The 10 ml samples were analyzed (including pH, specific gravity, and leukocytes) using an automatic urine analyzer, and the 50 ml samples were analyzed by DNA extraction, 16S rRNA gene amplification, and high-throughput sequencing. The correlation between routine urine analysis and sequencing results was also analyzed. Testing using the DESeq2 method showed that, at the order level, there were significant differences in the abundance of Caulobacterales between the urinary flora of the two groups (*P* < 0.05); family level, *Bacteroidaceae*, *Actinomycetaceae*, and *Tsukamurellaceae* (*P* < 0.05); genus level, *Finegoldia*, *Varibaculum*, *Actinobaculum*, *Propionimicrobium*, *Bacteroides*, *Brevundimonas*, and *Tsukamurella* (*P* < 0.05). LEfSe analysis found specific bacteria at the genus level in the urinary flora of the tumor group, namely, *Finegoldia* (genus Digestiflora) (*P* < 0.001) and Varibaculum (*P* < 0.001). Further correlation analysis showed that both species were positively correlated with the urine pH (*P* < 0.05). PICRUSt analysis showed significant differences in the two functional pathways of cell transformation and metabolism (*P* < 0.05). Combined with the results of bioinformatics analysis, some differential bacteria may be new biomarkers for urologic tumors, and there may be a correlation between urine pH and tumor occurrence. However, large-scale prospective studies and in vitro and in vivo experiments are required to further test and verify these findings.

## 1. Introduction

Malignant urinary tract tumors, including renal, ureteral, bladder, and prostate cancers, have high morbidity and mortality. The main causes of death are tumor invasion and metastasis to distant organs. Because the formation and development of tumors is a complex process, they exist in a vast and complex network and involve many factors. It is difficult to develop an effective method for completely eradicating malignant tumors. Epidemiological studies have shown that the incidence of urinary tract tumors is on the rise globally, which not only seriously affects the quality of life of patients but also threatens their lives. Early detection can significantly improve a patient's quality of life. Recent studies have shown that the occurrence and development of tumors are closely related to the tumor microenvironment (TME) [1]. The TME refers to the complex microecological internal environment in which tumors occur and develop and is another major feature of tumors in addition to the six major features. The TME is mainly composed of tumor cells, the extracellular matrix, lymphocytes, vascular-related endothelial cells, immune cells, fibroblasts, extracellular factors, and chemokines. These components are interrelated, affect each other, and play a role in the occurrence and development of tumors. The main features of the TME are related to chronic inflammation, environmental pH, immunosuppression, and oxygen supply. In recent years, some researchers have pointed out that gene mutations caused by changes in the microenvironment in which cells live are an important cause of tumorigenesis [2]. Urine is directly related to the urinary system: it is produced by the kidneys and excreted through the ureters, bladder, and urethra. Balance of the urine microecology is crucial for the health of the urinary system. Changes in the urine microenvironment and pH may be closely related to the formation of tract tumors. With the application of high-throughput sequencing technologies, such as 16S rRNA gene sequencing [3], an increasing amount of evidence has proved the existence of normal human urinary tract microflora. The urinary tract microbiota forms the urinary tract microecology with the host and the environment. The microecological imbalance of urinary tract flora is closely related to the occurrence and development of diseases, but there is still a lack of correlation research on the characteristics of urinary flora. Therefore, it was further proposed that the association between urinary system tumors and urinary tract microbes could be studied by analyzing the urine microflora. This study is aimed at further exploring the possible relationship between urinary flora and urinary tract tumors by comparing the characteristics of urinary flora between patients with urinary tract tumors and healthy individuals. Furthermore, the study sought to identify potential biomarkers and new targets for the prevention of urinary tumors.

## 2. Materials and Methods

### 2.1. Object of Study

#### 2.1.1. General Information

Ten cases were included in this study: the tumor group (average age, 66.00 ± 4.98 years), 6 cases of urological tumors that were treated in our hospital in 2020, and the health check-up (control) group (average age, 59.75 ± 3.30 years), 4 cases that came from the medical examination center. There were eight men and two women, and there was no significant difference in age and sex between the two groups. The study was approved by the ethics committee, and the patients signed informed consent forms.

#### 2.1.2. Entry Criteria

The relevant diagnostic criteria in *Campbell's Urology* (11th edition) were used in the tumor group, and all cases had a pathological diagnosis. The control group had no history of underlying diseases, and the patients were not currently taking any drugs.

#### 2.1.3. Exclusion Criteria

1. History of urinary tract infection in the past 1 month
2. History of antibiotic use in the past 1 month
3. History of transurethral procedures (catheterization, cystoscopy, cystostomy, and urodynamic examination) in the past 1 week

#### 2.1.4. Elimination Criteria

1. Specimen DNA quality inspection Grades C and D
2. Specimens suspected of contamination during sequencing
3. Samples with too few sequences obtained after sequencing

### 2.2. Research Methods

#### 2.2.1. Sample Collection

Samples (10 ml and 50 ml) of clean midsection urine were collected from both groups. The details are as follows:

1. 10 ml clean midsection urine sample: an automatic urine analyzer was used to conduct routine urinalysis on the 10 ml urine sample, and the analysis included pH, specific gravity, protein, sugar, red blood cells, and white blood cells
2. 50 ml clean midsection urine sample: after collection, the 50 ml urine sample was immediately stored in a refrigerator at 4°C and centrifuged (14000 rpm for 20 min) within 2 hours. The supernatant was discarded, and the precipitate was stored at -80°C for DNA extraction

#### 2.2.2. DNA Extraction and PCR Amplification

Microbial DNA was extracted from the samples using the E.Z.N.A.® Soil DNA Kit (Omega Bio-tek, Norcross, GA, USA) according to the manufacturer's protocol. The final DNA concentration and purification were determined using a NanoDrop 2000 UV-vis spectrophotometer (Thermo Scientific, Wilmington, USA), and the DNA quality was checked by 1% agarose gel electrophoresis. The V3-V4 hypervariable regions of the bacterial 16S rRNA gene were amplified using the primers 338F (5′-ACTCCTACGGGAGGCAGCAG-3′) and 806R (5′-GGACTACHVGGGTWTCTAAT-3′) using a PCR thermocycler (GeneAmp 9700, ABI, USA). The PCR reactions were conducted using the following program: 3 min of denaturation at 95°C; 27 cycles of 30 s at 95°C, 30 s for annealing at 55°C, and 45 s for elongation at 72°C; and a final extension at 72°C for 10 min. PCR reactions were performed in triplicate in a 20 *μ*l mixture containing 4 *μ*l of 5x FastPfu Buffer, 2 *μ*l of 2.5 mM dNTPs, 0.8 *μ*l of each primer (5 *μ*M), 0.4 *μ*l of FastPfu Polymerase, and 10 ng of template DNA.

#### 2.2.3. Illumina MiSeq Sequencing

The PCR products were recovered using a 2% agarose gel, purified using an AxyPrep DNA Gel Extraction Kit (Axygen Biosciences, Union City, CA, USA), eluted with Tris-HCl, and detected by 2% agarose gel electrophoresis. QuantiFluor™-ST (Promega, USA) was used for detection and quantification. The purified amplified fragments were constructed into a PE 2 × 300 library according to the standard operating procedures of the Illumina MiSeq platform (Illumina, San Diego, USA). Sequencing was performed on the MiSeq PE300 platform (Illumina) [4, 5].

#### 2.2.4. Bioinformatics Analysis

The original sequencing data were first filtered, and further operational taxonomic unit (OTU) clustering was performed. The species information abundance spectrum of OTUs and other species classifications were then formed [6, 7]. The species annotation information was obtained using QIIME software (http://qiime2.org/) [8], and the bacteria (biomarkers) with significant differences were identified by LEfSe analysis combined with the DESeq2 test [9, 10], and their influence was analyzed by linear discriminant analysis (LDA). The R language pheatmap package was used to draw the correlation heatmap [11]. The correlation heatmap can be used to analyze whether there is a significant correlation between environmental factors or other clinical phenotypic data and microbial communities or species. Furthermore, it can calculate the Spearman correlation coefficient between environmental factors or clinical phenotypic data and microbial species and display it with the heatmap. By providing data on environmental factors, such as pH, temperature, and clinical test results, species significantly related to a certain disease can be analyzed. The functional pathway of floral differences was predicted using the PICRUSt analysis tool [12]. The sequencing and bioinformatics services used in this study were completed by Microeco Tech Co. Ltd., Shenzhen, China.

#### 2.2.5. Statistical Methods

Data were analyzed using SPSS 22.0 (IBM Corp., Armonk, N.Y., USA), R software (V3.0.3), and QIIME (V2.0). If the measurement data obeyed a normal distribution, a *t*-test was used and expressed by $\bar{x}\pm s$, and *P* < 0.05 was considered statistically significant.

## 3. Results

### 3.1. Comparison of General Conditions between the Tumor and Control Groups

We compared demographic variables, such as BMI, smoking, alcohol consumption, diet, hyperlipidemia, diabetes, and hypertension, between the tumor and control groups (Table 1).

### 3.2. Analysis of Species Shared between the Two Groups

**Figure 1:**
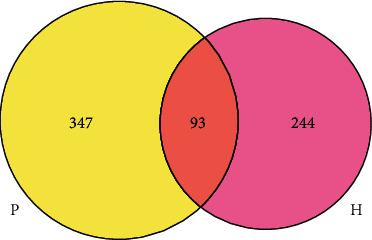
Number of common or unique species OTUs between the two groups. Note: the Venn diagram shows the number of common or unique OTUs between different groups, and each ellipse represents a group. In the figure, pink and yellow represent the normal and tumor groups, respectively. The number of OTUs contained in all samples is represented by the number of overlapping areas between the circles, and the number of unique OTUs in the sample is represented by the number of nonoverlapping areas.

A Venn diagram was used to analyze the unique or shared OTUs between the different sample groups. It can more intuitively display the number of shared and unique OTUs in different sample groups and can clearly show the overlap and composition similarity at the OTU level. There were differences in the composition and abundance of urinary flora between the normal and tumor groups (Figure 1), and the number of bacterial OTUs shared between the two groups was 93. The numbers of bacterial OTUs unique to the normal and tumor groups were 244 and 347, respectively.

### 3.3. Comparison of the Relative Abundance of Urine Microflora at Each Level between the Tumor and Control Groups

**Figure 2:**
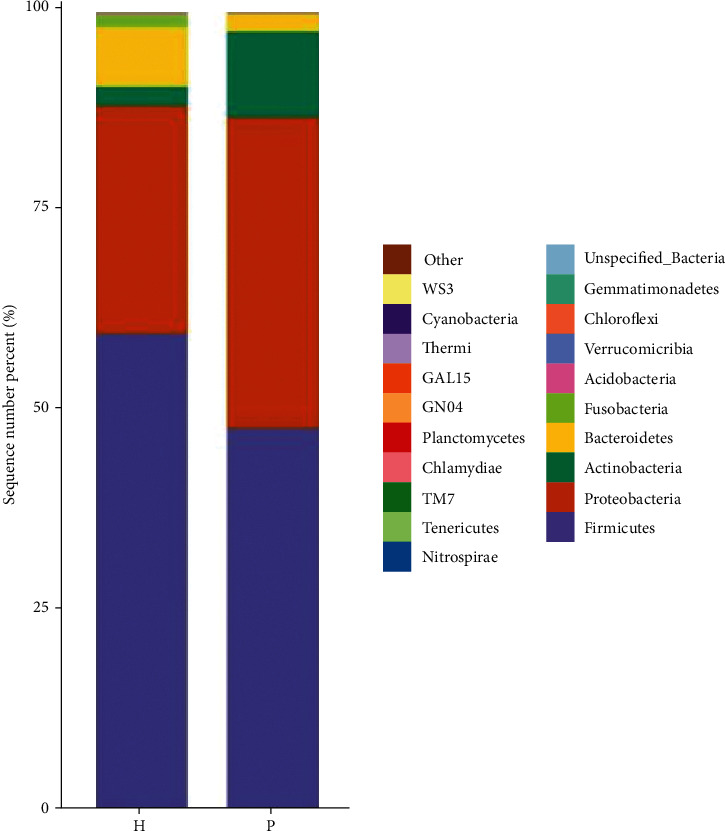
Histogram of the relative microbial distribution in the two groups at the phylum level. Note: the figure is a histogram of the relative distribution of the two groups at the phylum level (the top 20 species in relative abundance). The abscissa is the group name, the ordinate (sequence number percent) represents the ratio of the number of sequences annotated to this level to the total annotated data, and the top-down color order of the histogram corresponds to the color order of the legend on the right. The most dominant 20 species are shown in the legend, and the remaining species with lower relative abundances are classified and shown in the figure as “other.”

Figure 2 shows the relative abundance of urinary flora at the phylum level in the tumor and control groups. According to species annotation analysis at the phylum level, the dominant strains in the tumor group were *Firmicutes*, *Proteus*, *Actinomycetes*, and *Bacteroidetes*, in order of relative abundance (Figure 2 and Table 2). There was no significant difference between the two groups (*P* > 0.05).

**Figure 3:**
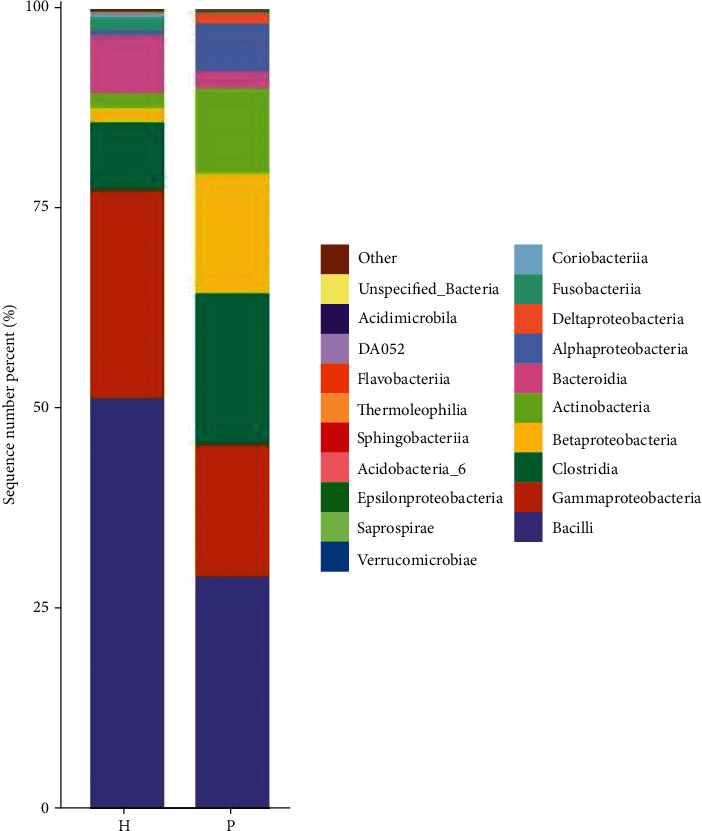
Histogram of the relative microbial distribution in the two groups at the class level (the top 20 species in relative abundance). Note: the figure is a histogram of the relative distribution of the two groups at the class level (the top 20 species in relative abundance). The abscissa is the group name, the ordinate (sequence number percent) represents the ratio of the number of sequences annotated to this level to the total annotated data, and the top-down color order of the histogram corresponds to the color order of the legend on the right. The most dominant 20 species are shown in the legend, and the remaining species with lower relative abundances are classified and shown in the figure as “other.”

At the class level, the dominant bacterial species in the tumor group were Bacilli, Clostridia, Gammaproteobacteria, Betaproteobacteria, Actinobacteria, Alphaproteobacteria, and Bacteroidia, in order of relative abundance (Figure 3 and Table 3). There was no significant difference between the two groups (*P* > 0.05).

**Figure 4:**
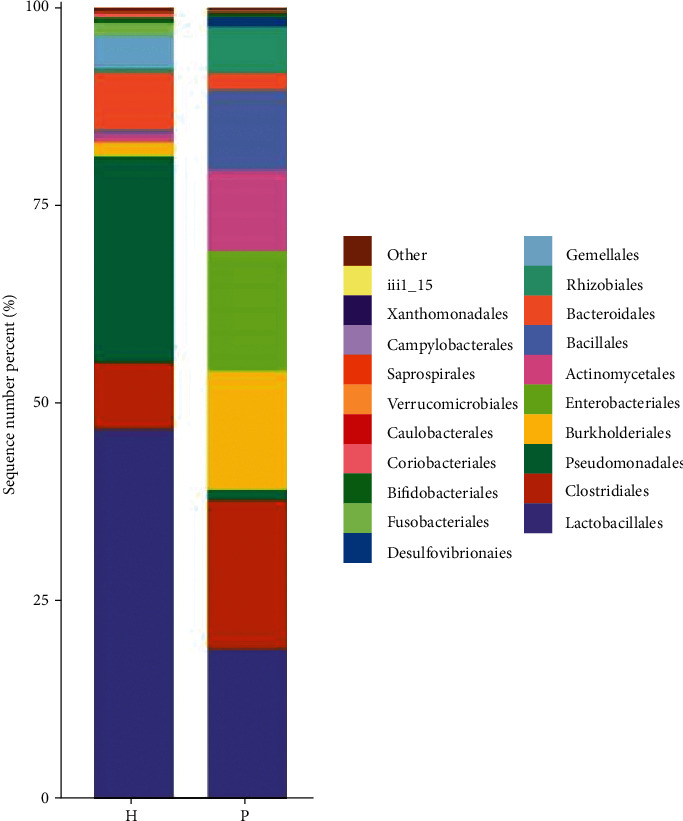
Histograms of the relative distribution of the two groups at the order, family, and genus levels, respectively (the top 20 species in relative abundance). The abscissa is the group name, the ordinate (sequence number percent) represents the ratio of the number of sequences annotated to this level to the total annotation data, and the top-down color order of the histogram corresponds to the color order of the legend on the right. The most dominant 20 species are shown in the legend, and the remaining species with lower relative abundances are classified and shown in the figure as “other.”

At the order level, using the DESeq2 method, there were significant differences in the abundance of Caulobacterales (*P* = 0.02) between the urinary flora of the two groups (Figure 4).

**Figure 5:**
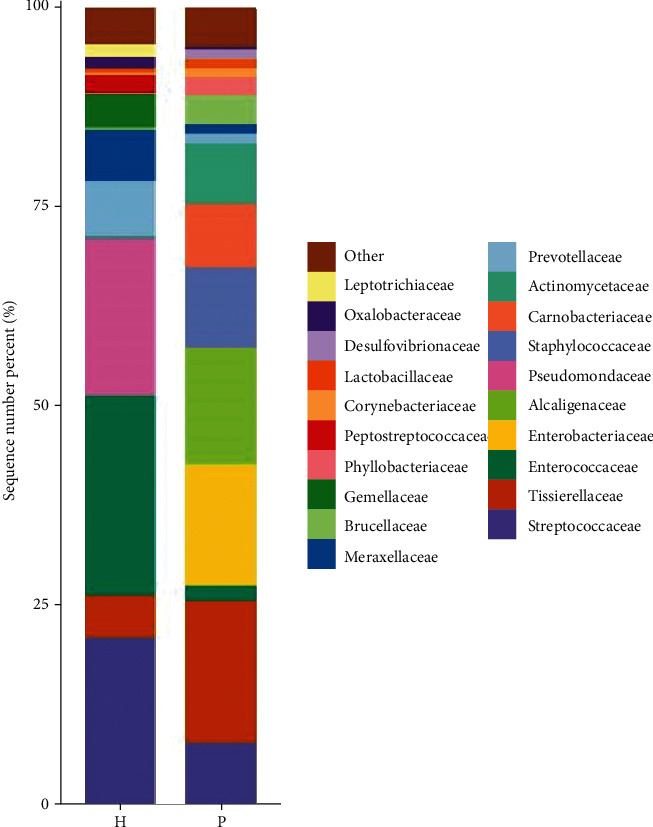
Histograms of the relative distribution of the two groups at the order, family, and genus levels, respectively (the top 20 species in relative abundance). The abscissa is the group name, the ordinate (sequence number percent) represents the ratio of the number of sequences annotated to this level to the total annotation data, and the top-down color order of the histogram corresponds to the color order of the legend on the right. The most dominant 20 species are shown in the legend, and the remaining species with lower relative abundances are classified and shown in the figure as “other.”

At the family level, as shown in Figure 5, using the DESeq2 method, there were significant differences in the abundance of *Bacteroidaceae* (*P* = 0.004), *Actinomycetaceae* (*P* = 0.04), and *Tsukamurellaceae* (*P* = 0.04) between the urinary flora of the two groups.

**Figure 6:**
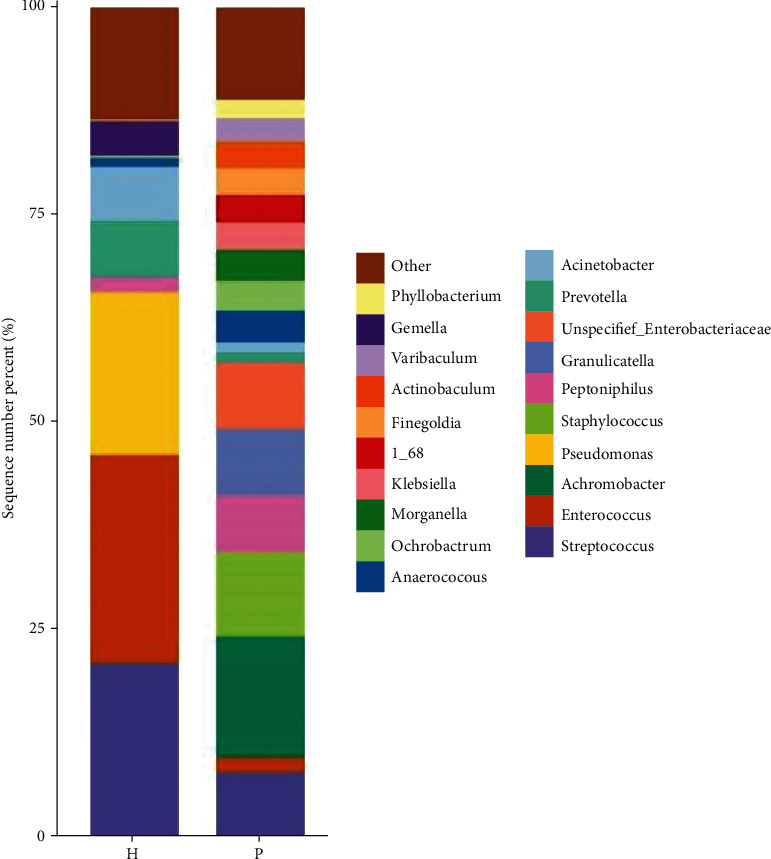
Histograms of the relative distribution of the two groups at the order, family, and genus levels, respectively (the top 20 species in relative abundance). The abscissa is the group name, the ordinate (sequence number percent) represents the ratio of the number of sequences annotated to this level to the total annotation data, and the top-down color order of the histogram corresponds to the color order of the legend on the right. The most dominant 20 species are shown in the legend, and the remaining species with lower relative abundances are classified and shown in the figure as “other.”

At the genus level, using the DESeq2 method, there were significant differences in the abundance of *Finegoldia* (*P* = 0.0001), *Varibaculum* (*P* = 0.0003), *Actinobaculum* (*P* = 0.002), *Propionimicrobium* (*P* = 0.004), *Bacteroides* (*P* = 0.004), *Brevundimonas* (*P* = 0.03), and *Tsukamurella* (*P* = 0.04) between the urinary flora of the two groups (Figure 6).  Table 4 shows the comparison of the abundance of microbial urinary tract flora at the order, family, and genus levels.

### 3.4. Specific Bacteria Associated with Urinary Tract Tumors

**Figure 7:**
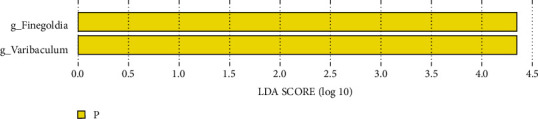
Linear discriminant analysis (LDA) column chart of LEfSe analysis at the genus level. Note: each horizontal column represents a species, and the length of the column corresponds to the LDA value. The higher the LDA value, the greater the difference. The color of the column corresponds to the group of characteristic microorganisms to which the species belongs, and the characteristic microorganisms (biomarkers) indicate the relatively abundant species in the corresponding group. The threshold in this figure was set at 4.0.

The LEfSe method is a combination of nonparametric tests and linear discriminant analysis and is suitable for testing differences in flora abundance. The LEfSe method was used to identify specific bacterial genera associated with urinary system tumors. At the genus level, there were specific bacterial genera in the tumor group, and the following two bacterial groups were isolated: *Finegoldia* and *Varibaculum* (Figure 7).

Further application of the DESeq2 test showed that *Finegoldia* (tumor group vs. control group, *P* = 0.0001) and *Varibaculum* (tumor group vs. control group, *P* = 0.0003) were statistically significant.

### 3.5. Correlation between Specific Bacteria and Urine pH

The correlation heatmap can be used to analyze whether there is a significant correlation between environmental factors, clinical phenotype data, and microbial communities or species. According to the analysis results, the Spearman correlation coefficient between the environmental factors and microbial species can be further calculated and displayed as a heatmap. By providing data on environmental factors, this analysis can identify species that are significantly associated with the disease. The environmental factors provided in this study were clinical phenotype data, including urinalysis and blood analysis data. Urinalysis data include urine pH and urine specific gravity (SG), and blood analysis data include white blood cells, neutrophils (N), alanine aminotransferase (ALT), serum creatinine (Cr), and blood urea nitrogen (BUN).

**Figure 8:**
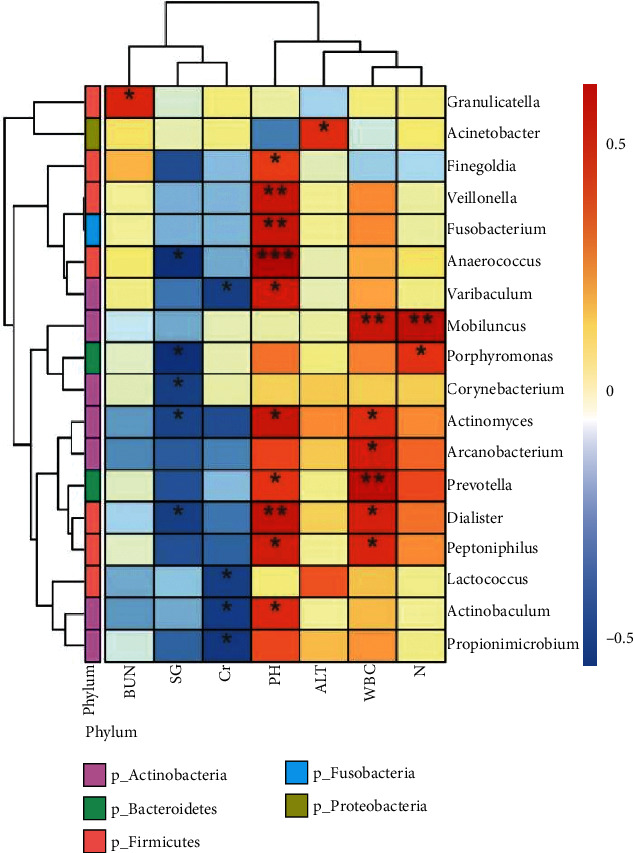
Heatmap of the correlation between microbial species and phenotype at the genus level. Note: environmental factors are on the *X*-axis and the species on the *Y*-axis. *R* values (rank correlation) and *P* values were calculated. The *R* values are displayed in different colors in the figure. If the *P* value is less than 0.05, it is marked with an ∗. The legend on the right shows the color intervals of the different *R* values. At the same time, the color bar on the left indicates the phylum classification of the species. ^∗^0.01 ≤ *P* < 0.05,  ^∗∗^0.001 ≤ *P* < 0.01, and^∗∗∗^*P* < 0.001.

The two specific species, *Finegoldia* and *Varibaculum*, were positively correlated with urine pH (Figure 8), and the difference was statistically significant (*P* < 0.05).

### 3.6. Potential Functional Pathways Associated with Urinary Tumors

**Figure 9:**
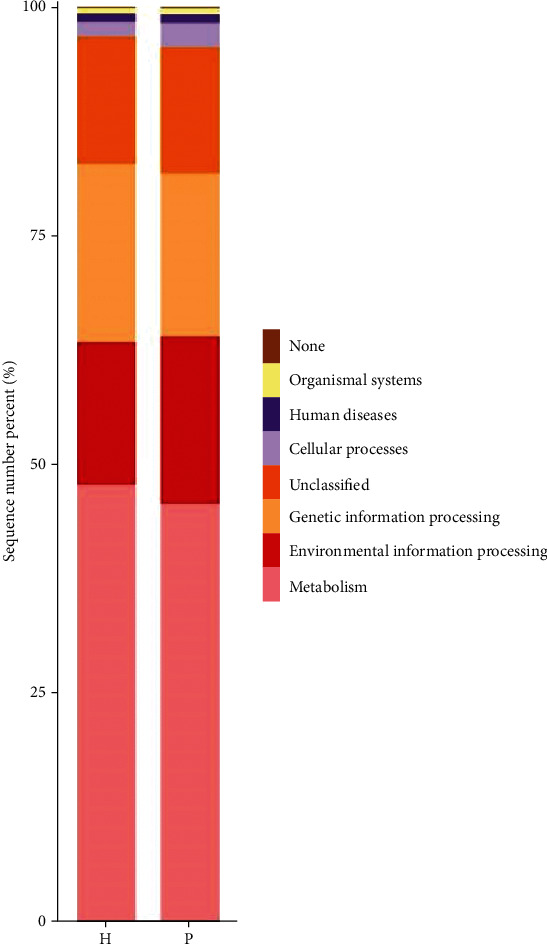
Histograms of the predicted composition of microbial community functions at the KEGG L1, L2, and L3 levels, respectively. KEGG results are displayed from top to bottom. The vertical axis represents the relative proportion annotated to a certain type of function; the horizontal axis represents the group name; the function category corresponding to each color block is shown in the legend on the right.

**Figure 10:**
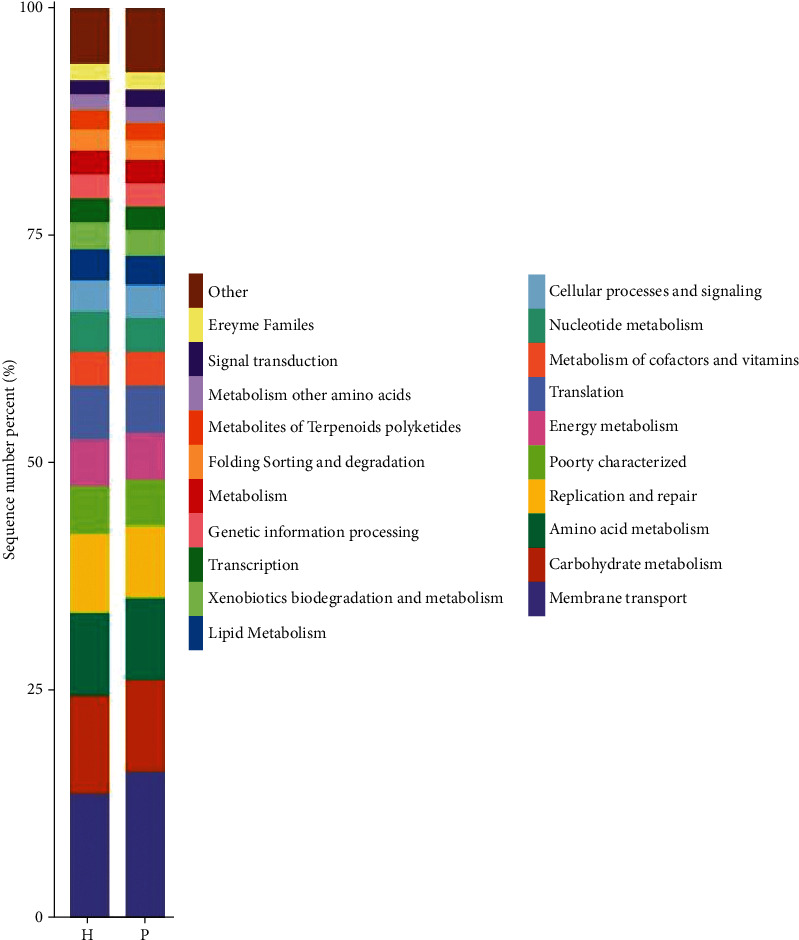
Histograms of the predicted composition of microbial community functions at the KEGG L1, L2, and L3 levels, respectively. KEGG results are displayed from top to bottom. The vertical axis represents the relative proportion annotated to a certain type of function; the horizontal axis represents the group name; the function category corresponding to each color block is shown in the legend on the right.

**Figure 11:**
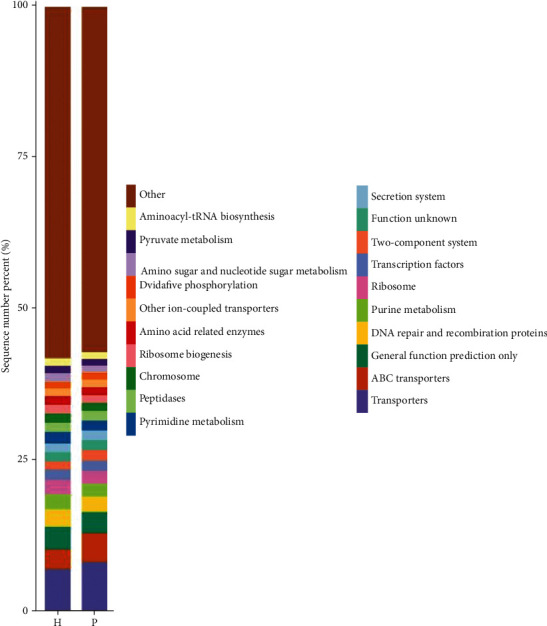
Histograms of the predicted composition of microbial community functions at the KEGG L1, L2, and L3 levels, respectively. KEGG results are displayed from top to bottom. The vertical axis represents the relative proportion annotated to a certain type of function; the horizontal axis represents the group name; the function category corresponding to each color block is shown in the legend on the right.

PICRUSt analysis was used to predict the metabolic functions of the flora. Figures 9–11 are column charts of the predicted composition of the microbial community function at the KEGG L1, L2, and L3 levels, respectively. The KEGG database (http://www.kegg.jp/) was subjected to KEGG enrichment analysis, and analysis of variance showed significant differences in two functional pathways, namely, the cell transformation (*P* = 0.03) and metabolism pathways (*P* = 0.04). The cell transformation pathway in the tumor group was higher than that in the control group, and the metabolic pathway was lower than that in the control group.

Upon further refining of the classification from L1 to L3, the cellular transformation pathway manifests as bacterial chemotaxis at the L3 level. Epithelial-mesenchymal transition (EMT) is a process of cell transformation in which the characteristics of epithelial cells are lost and those of mesenchymal cells are expressed by many regulatory factors through various regulatory pathways. Most solid tumors arise from epithelial cells throughout the body. Tumors are closely associated with long-term chronic inflammatory stimuli, and the inflammatory TME contributes to the malignant transformation of tumors. EMT in tumor cells depends on the participation of cytokines and chemokines in the microenvironment. The inflammatory microenvironment can induce and activate various pathways to promote tumor development, including EMT. In addition, an acidic microenvironment can promote EMT of tumor cells, which is conducive to tumor growth and metastasis.

The metabolic pathways were further refined into the metabolism of terpenes and polyketones, which were subsequently refined into the biosynthesis of polyketose units. Energy metabolism plays an important role in tumor occurrence and development. Abnormal cell microenvironments change the metabolic activity of tumor cells, and cell metabolism changes to support the synthesis of new proteins, lipids, and nucleic acids to ensure cell growth and split. These proteins are classified according to their roles in cellular metabolism, including the metabolic processes of sugars, lipids, energy, and amino acids. Regulation of cancer cell metabolism by regulating energy, lipids, amino acids, and protein synthesis enables better access to energy and nutrients from the TME. Low pH, hypoxia, and low glucose concentrations in the TME play an important role in tumor metabolism.

## 4. Discussion

Urinary tract tumors result from multiple internal and external factors. However, the specific mechanisms of action have not been fully elucidated. In recent years, multiple studies have shown that an imbalance in urinary flora is closely related to various urinary diseases, including urge incontinence [13], overactive bladder [14], interstitial cystitis [15], and diabetic bladder dysfunction [16]. The urinary tract flora is an important part of the urinary tract microenvironment. Therefore, further understanding of the characteristics of urinary flora may provide new ideas for research on the mechanisms and treatment of urinary tract tumors.

### 4.1. Urinary Flora May Become a New Biomarker for Urinary Tract Tumors

We analyzed the urinary flora characteristics of urinary tract tumors using 16S rRNA high-throughput sequencing. The Venn diagram shows that the urinary flora abundance of the tumor group was different from that of the control group. At the phylum level, the abundance of Proteobacteria and Actinobacteria in the tumor group was significantly higher than that in the control group, and that for Firmicutes and Bacteroidetes was significantly lower than that in the control group, but the difference was not statistically significant (*P* > 0.05). Using the DESeq2 method, we found that, at the order level, the abundance of Caulobacterales in the urinary flora of the two groups was significantly different (*P* = 0.02). At the family level, *Bacteroidaceae* (*P* = 0.004), *Actinomycetaceae* (*P* = 0.04), and *Tsukamurellaceae* (*P* = 0.04) showed significant differences in abundance between the urinary flora of the two groups. At the genus level, *Finegoldia* (*P* = 0.0001), *Varibaculum* (*P* = 0.0003), *Actinobaculum* (*P* = 0.002), *Propionimicrobium* (*P* = 0.004), *Bacteroides* (*P* = 0.004), *Brevundimonas* (*P* = 0.03), and *Tsukamurella* (*P* = 0.04) showed significant differences in abundance between the urinary flora of the two groups. Caulobacterales and Brevundimonas belong to the phylum Proteobacteria; *Actinomycetaceae*, *Tsukamurellaceae*, *Varibaculum*, *Actinobaculum*, *Propionimicrobium*, and *Tsukamurella* belong to Actinobacteria; *Bacteroidaceae* and *Bacteroides* belong to Bacteroidetes; and *Finegoldia* belongs to Firmicutes. Current studies have shown that most of the bacteria in the Actinomycetes and Proteobacteria phyla are pathogenic [17]. These include the Actinomycetes *Mycobacterium tuberculosis* and *Mycobacterium leprae*. Proteobacteria include *Escherichia coli*, *Salmonella*, and well-known species, such as *Vibrio cholerae* and *Helicobacter pylori*. The phylum Proteobacteria is the largest among bacteria. In recent years, an increasing number of studies have identified Proteobacteria as microbial markers of dysbacteriosis [18]. Many studies have shown that an increase in the number of members of Proteobacteria is a potential microbial feature of disease occurrence and is related to inflammatory factors [19].

Complicated interactions are formed between the urethral flora and the host, as well as within the flora, to ensure the stability of the microbial ecosystem. In a pathological state, the imbalance of the urethral microecology leads to the weakening of the urethral mucosal barrier function, which causes the urethral flora to be disordered. The decrease in the abundance of beneficial bacteria and increase in the abundance of harmful bacteria are its main characteristics. The disorder of the group in turn further affects the immune function of the urethral mucosa, triggering nonspecific inflammation of the urethra, and its inflammatory response further aggravates the damage and promotes tumor formation [20].

We further used the LEfSe method to identify specific bacterial genera related to urinary tract tumors in the tumor group at the genus level, namely, *Finegoldia* and *Varibaculum*. *Finegoldia* is a Gram-positive obligate anaerobe that is an opportunistic pathogen. Immunosuppression and malnutrition are currently recognized causes of symbiotic bacteria [21]. Recent studies [22] have found that some virulence factors related to the pathogenicity of *Finegoldia* bacteria include the L protein, peptostreptococcal albumin binding protein (PAB), SufA, and *Finegoldia* bacteria. Bacteria have the ability to form biofilms, which can evade host immune defense and antimicrobial treatment [23]. The pathogenic factors of *Varibaculum* are mainly related to enterotoxins, cytotoxins, endotoxins, adhesion, and colonization ability, which mainly cause human intestinal infections and various extraintestinal infections [24].

We used the PICRUSt analysis method to predict the metabolic function of the flora and found that the cell transformation pathway of the tumor group was higher than that of the control group, and the metabolic pathway was lower than that of the control group. The cell transformation pathway is mainly through the movement of cilia or flagella, which is manifested by chemotaxis of bacteria. Basic experiments have shown increased cell proliferation and cytokine or chemokine secretion in infected cells, which promotes immune escape and destroys the epithelial barrier, thereby inducing inflammation and leading to the occurrence and development of diseases [25].

Inflammation and tumors are closely related. Inflammatory reactions can stimulate the occurrence and development of tumors, and inflammation caused by tumors can cause gene mutations and malignancy [26]. The abundance of proinflammatory bacteria in the tumor group was relatively high, and the existing specific bacterial genera are related to the induction of inflammation and play a certain role in the occurrence of urinary system tumors [27]. These different bacterial genera may serve as new biomarkers for urinary system tumors. This provides a further reference for prognostic risk stratification of urinary system tumors.

### 4.2. Urine pH May Be Related to the Occurrence of Tumors

Changes in the urine microenvironment and pH can induce and activate the development of tumors. We identified specific bacterial genera, namely, *Finegoldia* and *Varibaculum*, in the tumor group using the LEfSe method. Furthermore, we conducted a correlation analysis, and the results showed that these two specific bacteria were positively correlated with urine pH (*P* < 0.05), and the difference was statistically significant. Tumor growth is closely related to the epidermal growth factor (EGF) in urine, and the EGF receptor (EGFR) is highly expressed in tumor cells [28]. The EGF is a low-molecular-weight peptide compound, composed of 53 amino acids, which promotes the proliferation of epithelial tissue. It exerts its physiological effects by specifically binding to the EGFR on the target cell membrane. The EGFR is regulated by the c-erbB-1 gene in the Src family of protooncogenes and is highly expressed in many tumors. As a normal component of the human body, the EGF concentration is highest in urine at approximately 25–250 ng/ml, which is dozens of times the blood concentration. The EGF is mainly produced by renal tubular epithelial cells in the kidneys [29, 30]. The binding of the EGF and EGFR is affected, to a certain extent, by the pH of the environment. Therefore, pH is an important factor affecting the binding of the EGF to its receptor [31]. Urine pH gradually increases from the renal pelvis to the bladder, and when the pH value is increased from 5.0 to 7.5, the binding rate of the EGF to its receptor increases 20 times. The increase in pH greatly increases the affinity of the EGF and EGFR and increases the growth-promoting effect of the EGF in urine on tumors. Therefore, there may be a correlation between urine pH and tumor occurrence.

However, this study had a small sample size and some shortcomings.

## 5. Conclusions

Symbiotic microorganisms play an important role in the human microenvironment. Their interactions with the host are comprehensive and extensive, and together they maintain the balance of the human microenvironment. Microecological imbalances are closely related to the occurrence and development of diseases. In this study, we discuss the possible carcinogenic effects of urinary tract bacteria and other microorganisms. The presence of specific bacteria may serve as a new biomarker for urinary tract tumors. Proinflammatory microorganisms and chronic inflammation mediated by an imbalance of the flora play a role in the occurrence of urinary system tumors. There may be a correlation between urine pH and tumor occurrence. Large-scale, large-sample prospective studies and in vivo and in vitro experiments are needed to further explore the relationship between urinary flora and urinary tumors.

## Figures and Tables

**Table 1:** Comparison of general characteristics between the two groups.

	Normal group (*n* = 4)	Tumor group (*n* = 6)
Sex		
Male	3 (30%)	5 (50%)
Female	1 (10%)	1 (10%)
Age	59.75 ± 3.30	66.00 ± 4.98
BMI (kg/m^2^)	28.84 ± 1.29	20.54 ± 3.54
Smoking status		
Smoker	1 (25%)	0 (0%)
Ex-smoker	1 (25%)	3 (50%)
Nonsmoker	2 (50%)	3 (50%)
Drinking status		
Drinker	0 (0%)	0 (0%)
Abstainers	0 (0%)	0 (0%)
Nondrinker	4 (100%)	6 (100%)
Dietary status		
High sodium diet	1 (25%)	2 (33.3%)
High fat diet	2 (50%)	2 (33.3%)
High sugar diet	1 (25%)	2 (33.3%)
Concomitant disease		
Hyperlipidemia	0 (0%)	2 (33.3%)
Diabetes	0 (0%)	2 (33.3%)
Hypertension	0 (0%)	2 (33.3%)

**Table 2:** Comparison of the abundance of microbial urinary tract flora in the two groups of samples at the phylum level.

Phylum	Normal group	Tumor group	*P* value
Firmicutes	59.78%	47.95%	*P* > 0.05
Proteobacteria	28.54%	38.92%	*P* > 0.05
Actinobacteria	2.36%	10.71%	*P* > 0.05
Bacteroidetes	7.39%	2.17%	*P* > 0.05

**Table 3:** Comparison of the abundance of microbial urinary tract flora in the two groups of samples at the class level.

Class	Normal group	Tumor group	*P* value
Bacilli	51.37%	29.11%	*P* > 0.05
Clostridia	8.40%	18.84%	*P* > 0.05
Gammaproteobacteria	26.06%	16.54%	*P* > 0.05
Betaproteobacteria	1.83%	14.97%	*P* > 0.05
Actinobacteria	1.80%	10.67%	*P* > 0.05
Alphaproteobacteria	0.62%	6.03%	*P* > 0.05
Bacteroidia	7.26%	2.11%	*P* > 0.05

**Table 4:** Comparison of the abundance of microbial urinary tract flora at the order, family, and genus levels.

	*P* value	Phylum level
Order		
Caulobacterales	0.02	Proteobacteria
Family		
*Bacteroidaceae*	0.004	Bacteroidetes
*Actinomycetaceae*	0.04	Actinobacteria
*Tsukamurellaceae*	0.04	Actinobacteria
Genus		
*Finegoldia*	0.0001	Firmicutes
*Varibaculum*	0.0003	Actinobacteria
*Actinobaculum*	0.002	Actinobacteria
*Propionimicrobium*	0.004	Actinobacteria
*Bacteroides*	0.004	Bacteroidetes
*Brevundimonas*	0.03	Proteobacteria
*Tsukamurella*	0.04	Actinobacteria

Note: the genera that are meaningful at the order, family, and genus levels belong to the phylum level.

## Data Availability

The data used to support the findings of this study are available in Genbank: sequence data: SUB9924783.
